# Fluconazole Dosing for the Prevention of *Candida* spp. Infections in Hemato-Oncologic Pediatric Patients: Population Pharmacokinetic Modeling and Probability of Target Attainment Simulations

**DOI:** 10.3390/pharmaceutics17040488

**Published:** 2025-04-08

**Authors:** Arkadiusz Adamiszak, Katarzyna Derwich, Alicja Bartkowska-Śniatkowska, Krzysztof Pietrzkiewicz, Izabela Niewiadomska-Wojnałowicz, Andrzej Czyrski, William J. Jusko, Agnieszka Bienert

**Affiliations:** 1 Department of Pharmacology, Poznan University of Medical Sciences, 60-806 Poznan, Poland; agbienert@ump.edu.pl; 2 Doctoral School, Poznan University of Medical Sciences, 60-812 Poznan, Poland; 3 Department of Pediatric Oncology, Hematology and Transplantology, Poznan University of Medical Sciences, 60-572 Poznan, Poland; kderwich@ump.edu.pl (K.D.); izabela.niewiadomskawojnalowicz@ump.edu.pl (I.N.-W.); 4 Department of Paediatric Anaesthesiology and Intensive Therapy, Poznan University of Medical Sciences, 60-572 Poznan, Poland; asniatko@ump.edu.pl (A.B.-Ś.); kpietrzkiewicz@ump.edu.pl (K.P.); 5 Department of Physical Pharmacy and Pharmacokinetics, Poznan University of Medical Sciences, 60-806 Poznan, Poland; aczyrski@ump.edu.pl; 6 Department of Pharmaceutical Sciences, School of Pharmacy and Pharmaceutical Sciences, State University of New York at Buffalo, Buffalo, NY 14214, USA; wjjusko@buffalo.edu

**Keywords:** fluconazole, population pharmacokinetics, Monte Carlo simulations, probability of target attainment, *Candida* spp. prophylaxis

## Abstract

**Objectives**: A population pharmacokinetic (popPK) model was used to evaluate fluconazole dosing regimens for *Candida* spp. prophylaxis in hemato-oncologic pediatric patients. **Methods**: Data were collected from patients receiving 3–12 mg/kg of fluconazole once daily as a 0.5 or 1 h infusion. Fluconazole concentrations were determined using a validated HPLC-UV method. The popPK model employed non-linear mixed effects modeling using the FOCEI algorithm implemented in nlmixr2. Monte Carlo simulations and probability of target attainment (PTA) analysis were performed in the rxode2 package to investigate dosing recommendations. **Results**: Concentration time data from nine patients, aged 7 months to 18 years, with 35 samples, were described by a one-compartment model with first-order elimination and allometric scaling of body weight. Assuming a *Candida* spp. MIC = 2 mg/L and the ratio of the area under the unbound concentration–time curve at a steady state to the MIC (*f*AUC/MIC) ≥ 100 as the pharmacokinetic/pharmacodynamic (PK/PD) target, the standard dosing regimens reported in the Summary of Product Characteristics (SmPC) did not achieve the target for patients treated with doses < 6 mg/kg. **Conclusions**: Hemato-oncologic pediatric patients require increased fluconazole doses to attain therapeutic efficacy. These results warrant clinical validation and should be confirmed by assessing a larger number of patients.

## 1. Introduction

Fluconazole (FLU) is a triazole group antifungal drug widely used in clinical conditions and outpatient treatment [1,2,3]. In the pediatric population, FLU is indicated to treat invasive and mucosal candidiasis, cryptococcal meningitis, and prophylaxis of *Candida* spp. infections in immunocompromised and other high-risk patients [2,4,5].

An oral dose of FLU has ~90% bioavailability and a comparable plasma concentration as intravenous (IV) administration [4,6,7,8,9]. The volume of distribution (*V*) of FLU is similar to the total body water of adult patients (0.7 L/kg) [4,6,10,11]. In turn, in children aged 0.25–12 years, the *V* is 33% higher (0.95 L/kg) [10,11]. The plasma protein binding is relatively low and reported as 11–12% [4,6,8,10]. Approximately 80% of the administered FLU dose is eliminated unchanged in urine [4,8,10,12,13]. The half-life in adults is about 30 h and in children is about 22 h, perhaps necessitating higher doses in children [10,11].

The activity and efficacy of FLU are related to the ratio of the area under the free drug concentration–time curve (*f*AUC) over 24 h at a steady state to the pathogen’s minimum inhibitory concentration (MIC) [14,15,16]. The higher pharmacokinetic/pharmacodynamic (PK/PD) target was established as an *f*AUC/MIC ≥ 100 [2,13,16,17]. For *Candida* spp. considered susceptible to FLU treatment (MIC ≤ 2 mg/L) [18], the *f*AUC must reach at least 200 mg × h/L [2,15]. The less rigorous PK/PD target is fAUC/MIC ≥ 50; when assuming MIC ≤ 2 mg/L, the *f*AUC is thus at least 100 mg × h/L [17,19]. Although attaining the PK/PD targets improves treatment outcomes, therapeutic drug monitoring (TDM) is not a clinical routine mainly because of the broad FLU therapeutic index, favorable safety profile, and linear dose–concentration correlation [14,17,20,21,22]. Lack of routine TDM combined with limited knowledge of the PK of azoles in the pediatric population results in subtherapeutic FLU dosages in almost 40% of patients [3,14,15,17,22].

Since underexposure to FLU during treatment of the pediatric population has been suggested in the literature, our study aimed to investigate the efficacy of registered FLU doses used as a prophylaxis of *Candida* spp. infections.

## 2. Materials and Methods

### 2.1. Study Population and Data Collection

This study was performed in accordance with the Declaration of Helsinki, approved by the local ethics committee (the Poznan University of Medical Sciences Bioethics Committee, decision number: KB-820/21, 3 November 2021), and registered at ClinicalTrials.gov (NCT05426499). Written informed consent was obtained from patients and their legal representatives before inclusion. This study was conducted at the Poznan University of Medical Sciences (PUMS) Karol Jonscher Teaching Hospital: Department of Pediatric Oncology, Hematology and Transplantology, and Department of Paediatric Anaesthesiology and Intensive Therapy. Patients were recruited between December 2022 and October 2024.

Inclusion criteria for the study were age <18 years and treatment or prophylaxis with IV FLU administered according to the Summary of Product Characteristics (SmPC). The sampling procedure involved minimizing patient risk by collecting most of the blood samples at the time of scheduled biochemical assays (like complete blood count or CRP), routinely performed during hospitalization. Consequently, sampling times varied for all patients. Baseline demographic, biological, and clinical data were collected from medical patient records and included sex, age, body weight, height, body surface area (DuBois Method), the serum creatinine estimated glomerular filtration rate (eGFR) calculated using the Bedside Schwartz equation [23], eGFR calculated using the Schartz 2012 equation [24], bilirubin, total proteins, aspartate aminotransferase (AST), alanine aminotransferase (ALT), C-reactive protein (CRP), and procalcitonin (PCT).

### 2.2. Bioanalytical Methods

Blood samples (0.5 mL) were collected into lithium heparin tubes (BD Vacutainer^®^, Becton, Dickinson and Company, Franklin Lakes, NJ, USA) and then immediately centrifuged at 3000× *g* for 15 min to harvest plasma. Collected plasma samples were stored at −25 °C pending analysis. Fluconazole plasma concentrations were determined using our previously developed and validated method involving high-performance liquid chromatography with ultraviolet detection (HPLC-UV) [25]. The determination range was 0.5–100.0 mg/L.

### 2.3. Population Pharmacokinetic Analysis

Fluconazole concentration–time data were analyzed using PopPK modeling using the first-order conditional estimation with interaction (FOCEI) algorithm for non-linear mixed effects models implemented in the nlmixr2 R package [26]. One- and two-compartment structural compartment models with linear clearance (*Cl*) from the central compartment were tested. The interindividual variability (IIV) of PK parameters was assumed to follow a log-normal distribution. Proportional and combined error models were tested to describe the residual unexplained variability. Allometric scaling of the parameters was implemented according to the following:

(1) $$CL={CL}_{T}\times {\left(\frac{WT}{70}\right)}^{0.75}$$

and

(2) $$\;V=V_{T}\times {\left(\frac{WT}{70}\right)}^{1}$$

where *CL* and *V* are the clearance and volume of distribution, *CL_T_* and *V_T_* are typical values for a 70 kg adult, and *WT* is body weight expressed in kilograms.

The covariate model was investigated according to the classic stepwise covariate modeling method [27]. Pearson’s correlation test was performed to check for any relationships between random effects and covariates and whether they should be added to the model. Additionally, the mlcov R package was used to investigate possible covariates that should be considered during model development [28]. The effect of covariates was evaluated using the following equations.

For continuous covariates

(3) $$\theta_{i}=\theta_{pop}\times {\left(\frac{{COV}_{i}}{{median}_{{COV}_{i}}}\right)}^{\theta_{cov}}\times e^{\eta_{i}}$$

For categorical covariates

(4) $$\theta_{i}=\theta_{pop}\times e^{\theta_{cov}}\times e^{\eta_{i}}$$

where *θ_i_* represents the individual parameter estimate, *θ_pop_* is the population estimated value for this parameter, *COV_i_* corresponds to the individual value of a covariate, *θ_cov_* is the estimated effect of that covariate on the parameter, and *η_i_* is equal to the individual value of the random effect associated with the parameter describing the difference between the population value of the parameter and the individual value of that parameter for the *i*th subject.

Model selection was based on a decrease of at least 3.84 points (*p* < 0.05) for 1 degree of freedom in the Bayesian Information Criteria (BIC), objective function value (OFV), the stability of the model, the precision of the parameter estimates, and the goodness-of-fit (GOF) diagnostic plots evaluated at each step of the building process. The final model was assessed using Visual Predictive Checks (VPCs).

### 2.4. Simulations

Simulations of *f*AUC after the 6th dose (between 144 and 168 h) and dosing regimens based on the final model were performed in the rxode2 R package [29]. We tested 3–12 mg/kg in a 30 min infusion daily according to the dosages proposed in SmPC for prophylaxis of *Candida* spp. infections. Additionally, we tested alternatively higher 15 and 18 mg/kg doses. For each combination, 2500 virtual patients were simulated as 50 patients per group replicated 50 times, accounting for the same individuals among the simulated groups. An *f*AUC/MIC ≥ 100 was the PK/PD target, according to the European Committee on Antimicrobial Susceptibility Testing (EUCAST) [12,16]. Alternatively, a less rigorous PK/PD target (*f*AUC/MIC ≥ 50) was also investigated [17,19]. According to the EUCAST breakpoints for antifungals, we assumed an MIC = 2 mg/L for FLU as the highest MIC value of susceptible *Candida* spp. strains. [18]. The probability of target attainment (PTA) > 90% was considered an acceptable probability of success [30]. Assuming an FLU half-life of about 30 h and at least 5 half-lives to reach a steady state, for the PTA analysis we simulated the *f*AUC after the 6th dose (between 144 and 168 h) [4,18]. The free fraction of FLU was calculated based on the ~11% protein binding in the SmPC [4]. In addition, simulations for standard SmPC doses and the *f*AUC/MIC ≥ 100 PK/PD target with MIC values from 0.125 to 2 mg/L were performed.

## 3. Results

### 3.1. Study Population

A total of 35 plasma concentrations from nine patients were used. The average number of samples per patient was four, with a range of two and five. All concentrations were above the lower limit of quantification (0.5 mg/L) [25]. Patients received FLU in doses of 3–11 mg per kg of body weight as 0.5–1 h infusions once daily. The baseline characteristics of the studied population are presented in Table 1.

### 3.2. Population Pharmacokinetic Model

Plasma FLU concentrations are described by the one-compartment model with clearance from plasma. The allometric scaling, according to Equations (1) and (2), enabled the model to fit well. The proportional error model resulted in the best data fit. The final model estimates are presented in Table 2. The range of estimated *CL* values normalized for body weight is 0.31 mL/min/kg for a 58.5 kg patient to 0.55 mL/min/kg for a 6 kg patient. The estimated *V* value normalized for body weight is 1.49 L/kg.

**Figure 1:**
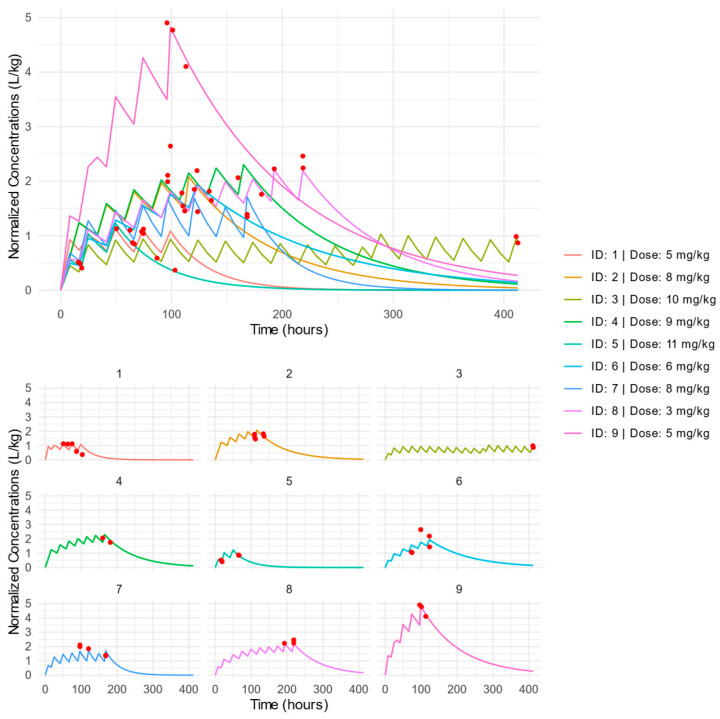
Clustered (**Top**) and separate (**bottom**) individual PK profiles of dose-normalized FLU concentrations versus time. Red dots indicate individual patient concentrations.

The individual PK profiles of dose-normalized concentrations in time are presented in Figure 1. Similar regular and log-scaled graphs clustered according to dose group are presented in Figures S1 and S2. The VPC and GOF plots for the final model indicated a good description of the data and no major model misspecification. The points in the observed vs. predicted plots are symmetrically clustered around the line, indicating no evident trends. The residuals are distributed around zero, and most points are within the range of −2 and 2 (Figure S3). The VPC showed that most observed concentrations were within the predicted intervals (Figure S4). The nlmixr2 code of the final model along with graphs of the distribution of the residuals (Figure S5), the distribution of the individual parameters (Figure S6), the distribution of the standardized random effects (Figure S7), and the correlation between random effects (Figures S8 and S9) are presented in the Supplementary Materials.

**Figure 2:**
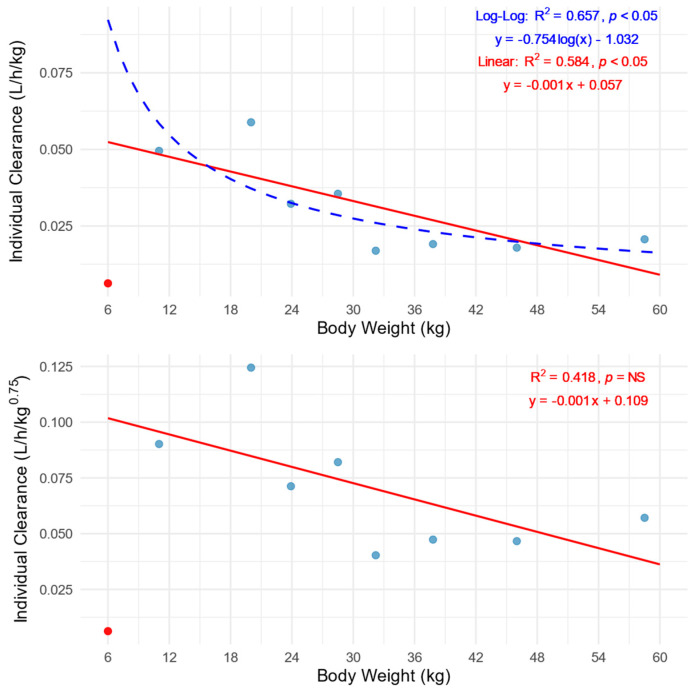
Relationship of body-weight-normalized clearances (*CL*/BW **top** and *CL*/BW^0.75^ **bottom**) versus patients’ body weight. The red lines represent linear regression analysis, while the blue dashed line corresponds to the log-log regression analysis. The blue dots indicate individual patients’ values, while the red dot represents an outlier patient ID: 9.

In accordance with the assumptions of allometric scaling, the values of clearance normalized by body weight significantly decrease with increasing body weight, which best explains the course of the log-log regression curve. In contrast, the lack of significant correlation when *CL* is normalized based on body weight to the power of 0.75 indicates the validity of using allometric scaling of *CL* according to Equation (1) and compensating for the effect of body weight on *CL* (Figure 2).

**Figure 3:**
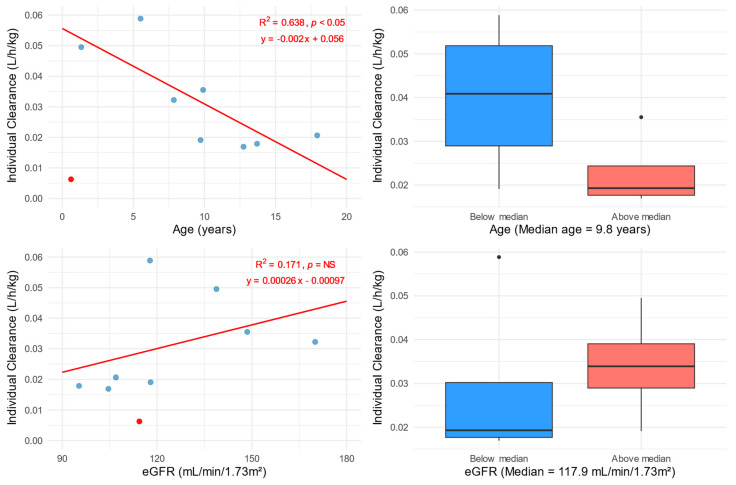
Total FLU clearance estimated from the popPK analysis and normalized for body weight versus age (**left top**) and calculated using the Schwartz 2012 equation eGFR (**left bottom**). Total FLU clearance estimated from the popPK analysis and normalized for body weight in relation to age group (**top right**) and eGFR group (**bottom right**). Patient ID: 9 was excluded from the analysis as an outlier (marked with a red dot). The blue dots indicate individual patients’ values. Solid red lines represent linear regressions with fit (R^2^), *p*-value, and equation values shown in the right corners of the graphs. The division into groups was based on the median of the analyzed variables.

A post hoc analysis of the effect of age on the *CL* of fluconazole showed a statistically significant decrease in *CL* with increasing age. The median *CL* for patients aged below the median age is 0.68 mL/min/kg, while for older patients it is 0.32 mL/min/kg. The effect of eGFR on fluconazole *CL* was not significant; however, differences in *CL* were observed according to an eGFR above or below the median. Patients with an eGFR below 117.9 mL/min/1.73 m^2^ showed a median *CL* of 0.32 mL/min/kg, while patients with a higher eGFR had a *CL* of 0.56 mL/min/kg (Figure 3).

### 3.3. Simulations and Probability of Target Attainment Analysis

**Figure 4:**
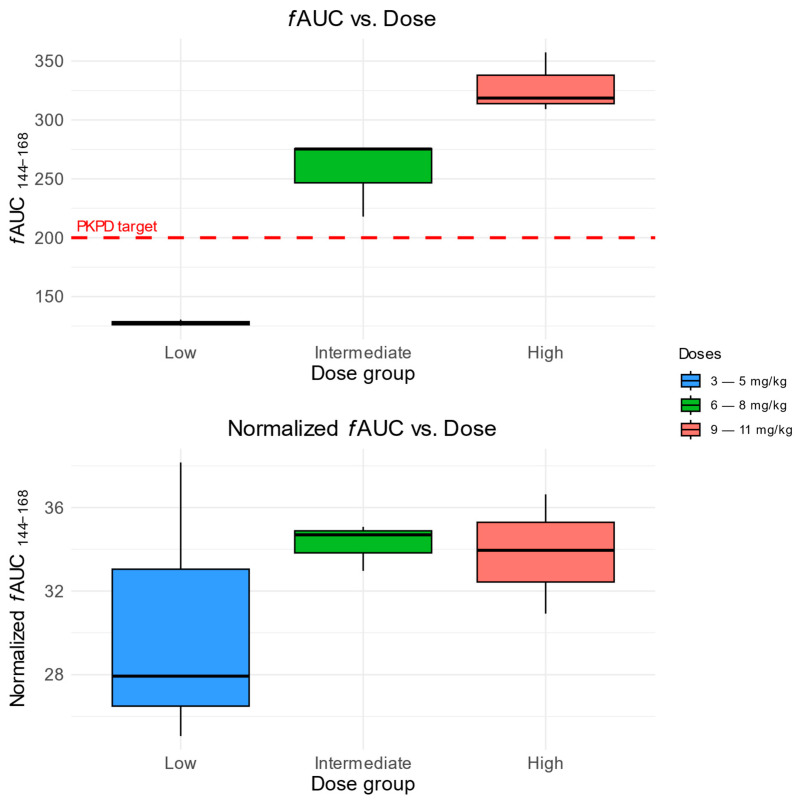
Predicted *f*AUC (**Top**) and dose-normalized *f*AUC (**Bottom**) in FLU dose groups. The red dashed line indicates the assumed PK/PD target (fAUC/MIC ≥ 100) for *Candida* spp. MIC = 2 mg/L.

The evaluation of the *f*AUC at a steady state indicates that children treated with low doses of FLU (3–5 mg/kg) were not able to achieve the intended therapeutic target (*f*AUC/MIC > 100, assuming *Canida* spp. MIC = 2 mg/L). Patients treated with intermediate (6–8 mg/kg) and high (9–11 mg/kg) FLU doses achieved an average of 256 and 328 mg × h × L^−1^ (Figure 4).

According to the dosing simulations, for the *f*AUC/MIC ≥ 100 target, already registered FLU dosages exceed the assumed 90% of PTA in the case of 48–60 kg patients treated with 12 mg/kg FLU dose. For the *f*AUC/MIC ≥ 50 target, the higher SmPC doses (9–12 mg/kg) enable the target PTA to be achieved. The simulation results for both PK/PD targets and MIC = 2 mg/L are presented in Figure S10. The PTA analysis of different MIC values of susceptible *Candida* spp. (0.125–2 mg/L) showed that at MIC values ≤ 1 mg/L, the FLU doses indicated in the SmPC are sufficient to achieve the target *f*AUC/MIC ≥ 100 (Figure S11).

## 4. Discussion

This may be one of the first FLU popPK models devoted to *Candida* spp. prophylaxis in hemato-oncologic pediatric patients. In addition to providing PK assessment, the PK model served as a tool to evaluate the effectiveness of FLU doses registered for the prophylaxis of *Candida* spp. infections. We found that the registered FLU doses < 6 mg/kg appear to be of limited value owing to resultant subtherapeutic concentrations. Consequently, higher doses (6–12 mg/kg) appear necessary in children.

The one-compartment model appeared adequate for our limited data. Adding a second compartment did not show a statistically significant improvement and even decreased the stability of the estimated parameters. The relatively small number of samples per patient and the unsystematized sample collection timing explained this. The opportunistic approach to sample collection limited sampling, and so increasing the number of recruited patients was not feasible in our study [31,32]. Nonetheless, given its implementation in most published FLU popPK models in different populations, the one-compartment model was sufficient to analyze FLU PK [2,7,15,30,33,34,35,36,37,38,39,40]. The estimated *CL* calculated per kg of body weight (0.018 L/h/kg for 70 kg adult) is consistent with previously published FLU pop models [30,34]. In turn, the estimated *V* per kg (1.49 L/kg) is higher (~50%) than published values for children or adults [3,30,34]. This may be a result of the underlying illness, administered chemotherapy, and/or hyper-hydration [14,41]. The increased *V* in pediatric oncology patients could explain the reduced FLU exposure observed in the Van Der Elst et al. study compared to other pediatric populations [14]. Nonetheless, the observed *V* needs to be confirmed in future FLU PK studies in similar patients.

The slope coefficient of log-log regression for the body-weight-normalized *CL* versus body weight equal to −0.754 is consistent with the assumptions of allometric scaling and the use of *CL* per kg scaling to the power of 0.75. Additional confirmation of the discussed assumption is the lack of a significant relationship between *CL*/BW^0.75^ and body weight (Figure 2). This is in line with existing knowledge of allometric *CL* scaling in the pediatric population [42,43].

In general, according to the SmPC and FLU Product Label, children have a higher FLU *CL* than adults [4,44]. This is consistent with our results, where patients, especially those younger than the median age, showed a *CL* equal to 0.65 mL/min/kg. In the case of pediatric patients older than the mentioned median (9.8 years), the *CL* was lower (0.32 mL/min/kg) and closer to the *CL* for adults (0.23 mL/min/kg) reported in the Product Label [44]. Some trends were observed in the relationship between the body-weight-normalized *CL* and eGFR (Figure 3). In general, patients with an eGFR lower than 117.9 mL/min/1.73 m^2^ (population median calculated using Schwartz et al. equation [24]) showed a lower *CL*/BW (0.32 mL/min/kg) than patients with a higher eGFR (0.56 mL/min/kg). The effect of the eGFR on *CL* is primarily due to the significant elimination of FLU via the renal route [4,44]. Indirect confirmation of our observation is also provided by previous PK FLU studies indicating serum creatinine as a covariate of *CL* [30,37,39,45].

The results of the *f*AUC simulations showed that low doses of FLU (3–5 mg/kg) are insufficient to achieve the intended PK/PD target and thus show unsatisfactory efficacy in the prophylaxis of *Candida* spp. infections. The PTA analysis results indicated the limited utility of the doses in the SmPC when it is necessary to achieve *f*AUC/MIC ≥ 100, assuming a *Candida* spp. MIC = 2 mg/L. In the case of a lesser PK/PD target (*f*AUC/MIC ≥ 50), the doses between 6 and 12 mg/kg were sufficient to achieve a PTA ≥ 90%. The need for higher dosages in the pediatric cancer patient population is consistent with the observations of Van Der Elst et al., resulting in recommendations for usage of 12 mg/kg doses in their hospital [14]. According to the PTA analysis, the MIC values > 2 mg/L make it impossible to achieve the PK/PD target (*f*AUC/MIC ≥ 100) with the FLU doses included in the SmPC. This observation is consistent with the data for *Candida* spp. susceptibility to FLU treatment presented in the latest “Breakpoint tables for interpretation of MICs for antifungal agents” established by the EUCAST [16,18].

The individual PK of patient ID: 9 stands out (Figure 1). The explanation most notably for the low *CL* (0.006 L/h/kg) was the patient’s young age of 6 months. This is supported by the Fluconazole Product Label and pharmacokinetic studies conducted in infants [3,30,44,46]. The median *CL* reported by Watt et al. for non-ECMO patients aged 31 days to 2 years was 0.017 L/h/kg with a range of 0.008 to 0.029 L/h/kg. Thus, patient ID: 9 was excluded from the fitting analysis and is presented in the graphs as a red dot (Figure 2 and Figure 3).

Our main limitation is the small number of patients and plasma samples per patient. This is due to difficulties in recruiting pediatric patients, which resulted in high refusal rates. A less critical limitation is the inability to determine the free fraction of FLU. However, binding to proteins at ~11% would have a minor impact on the PK of FLU. Given the above, we recommend caution in the clinical implementation of our study results. The raw data listed in the Supplementary Materials may help expand the knowledge of PK FLU in the search for optimal FLU therapy in the pediatric population.

## 5. Conclusions

Body weight implemented as allometric scaling influences the elimination of FLU, leading to the need for tailoring dosages to achieve the assumed PK/PD target for *Candida* spp. infection prophylaxis. As a rule, decreased weight (correlated with younger age) requires increased FLU doses per kg of body weight. Although the eGFR was not included as one of the covariates in the popPK model, indirect analysis confirmed its effect on fluconazole’s CL as observed in studies to date. In general, doses < 6 mg/kg pose a risk of subtherapeutic concentrations and, as a consequence, higher (6–12 mg/kg) doses are recommended for hemato-oncologic pediatric patients. In turn, even higher than registered FLU dosages might be required to reach an *f*AUC/MIC ≥ 100; however, its safety has not yet been studied. The described approach does not apply to *Candida glabrata*, for which the MIC determining susceptible strains is < 0.001 mg/L [18].

## Figures and Tables

**Table 1:** Patients’ characteristics (n = 9).

Characteristics	Number (%) or Median [Range]
Sex	
Female/Male (%)	6 (66.7%), 3 (33%)
Age (years)	9.75 [0.50–18.00]
Weight (kg)	28.50 [6.00–58.50]
Height (cm)	28.50 [74.00–178.00]
Body surface area, BSA (m^2^)	1.14 [0.35–1.73]
Serum creatinine, S_Cr_ (mg/dL)	0.31 [0.18−0.59]
Bedside Schwartz eGFR (mL/min/1.73 m^2^)	151.0 [115.5–240.3]
Schwartz 2012 eGFR (mL/min/1.73 m^2^)	117.77 [95.31–170.03]
Bilirubin (mg/dL)	0.50 [0.19–1.55]
Total proteins (g/dL)	5.70 [4.80–6.87]
Alanine aminotransferase, AST (IU/L)	26.0 [14.0–77.0]
Aspartate aminotransferase, ALT (IU/L)	44.0 [8.0–139.0]
C-reactive protein, CRP (mg/dL)	0.34 [0.02–6.71]
Procalcitonin, PCT (ng/mL)	0.30 [0.04–1.92]

**Table 2:** Estimates for the base and final population pharmacokinetic models for fluconazole.

Parameters	Mean Estimate (%RSE)
	A One-Compartment Model with Allometric Scaling (Final Model)
Clearance, *CL* (L/h)	1.24 (23.23)
Weight (kg), WT on *CL*	fixed 0.75
*IIV* on *CL* (CV%)	88.54
Volume, *V* (L)	104.07 (21.59)
Weight (kg), WT on *V*	fixed 1.00
*IIV* on *V* (CV%)	55.85
Proportional residual error (CV%)	25.19
BIC	208.82
OFV	126.72

BIC, Bayesian Information Criteria; CV%, coefficient of variation calculated as $\sqrt{\exp\left(\omega^{2}\right)-1}\times 100\%$; *IIV*, interindividual variability; OFV, objective function value.

## Data Availability

Data are contained within the article and Supplementary Materials.

## Supplementary Materials

The following supporting information can be downloaded at https://www.mdpi.com/article/10.3390/pharmaceutics17040488/s1, Figure S1: Dose-normalized concentrations for each dose group versus time; Figure S2: Dose-normalized concentrations (logarithmic scale) for each dose group versus time; Figure S3: Goodness-of-fit plots of the final fluconazole one-compartment model; Figure S4: VPC plot of the final fluconazole one-compartment model; Figure S5: Distribution of residuals; Figure S6: Model parameter distribution; Figure S7: Random effects plots; Figure S8: Correlations of random effects; Figure S9: Correlations of random effects excluding outlier (marked in red); Figure S10: Probability of target attainment charts for fluconazole dosages 3–18 mg/kg, body weight 6–60 kg, and *f*AUC/MIC ≥ 100 (top) or *f*AUC/MIC ≥ 50 (bottom) assuming *Candida* spp MIC = 2 mg/L; Figure S11: Probability of target attainment charts for fluconazole doses 3–12 mg/kg, body weight 6–60 kg, and *f*AUC/MIC ≥ 100, assuming different *Candid*a spp MICs; nlmixr2 final model code; GitHub repository hyperlink.

## Abbreviations

The following abbreviations are used in this manuscript:

ALT	Alanine aminotransferase
AST	Aspartate aminotransferase
BIC	Bayesian Information Criteria
BW	Body weight
*CL*	Clearance
*COVi*	Individual value of a covariate
CRP	C-reactive protein
CV%	Coefficient of variation
CWRES	Conditional weighted residuals
eGFR	Estimated glomerular filtration rate
EUCAST	The European Committee on Antimicrobial Susceptibility Testing
*f*AUC	Area under the free drug concentration–time curve
FLU	Fluconazole
FOCEI	First-order conditional estimation with interaction
GOF	Goodness of fit
HPLC-UV	High-performance liquid chromatography with ultraviolet detection
*IIV*	Interindividual variability
MIC	Minimum inhibitory concentration
OFV	Objective function value
PCT	Procalcitonin
PK	Pharmacokinetic
PK/PD	Pharmacokinetic/pharmacodynamic
popPK	Population pharmacokinetic
PTA	Probability of target attainment
SCr	Creatinine serum
SmPC	Summary of Product Characteristics
TDM	Therapeutic drug monitoring
*V*	Volume of distribution
VPC	Visual Predictive Checks
WT	Body weight
*ηi*	Random effect
*θcov*	Estimated effect of the covariate on the parameter
*θi*	Individual parameter estimate
*θpop*	Population estimated value for the parameter
