# Supplementary material for: Fluconazole Dosing for the Prevention of Candida spp. Infections in Hemato-Oncologic Pediatric Patients: Population Pharmacokinetic Modeling and Probability of Target Attainment Simulations
Source: Pharmaceutics. 2025 Apr 8;17(4):488. doi: 10.3390/pharmaceutics17040488 (PMC12030177; doi:10.3390/pharmaceutics17040488)
Supplement: Supplementary file 1 [file pharmaceutics-17-00488-s001.zip › pharmaceutics-3544017-supplementary.pdf]

Supplementary materials for "Fluconazole dosing for the prevention of *Candida* spp. infections in hemato-oncologic pediatric patients: a population pharmacokinetic modeling and probability of target attainment simulations."

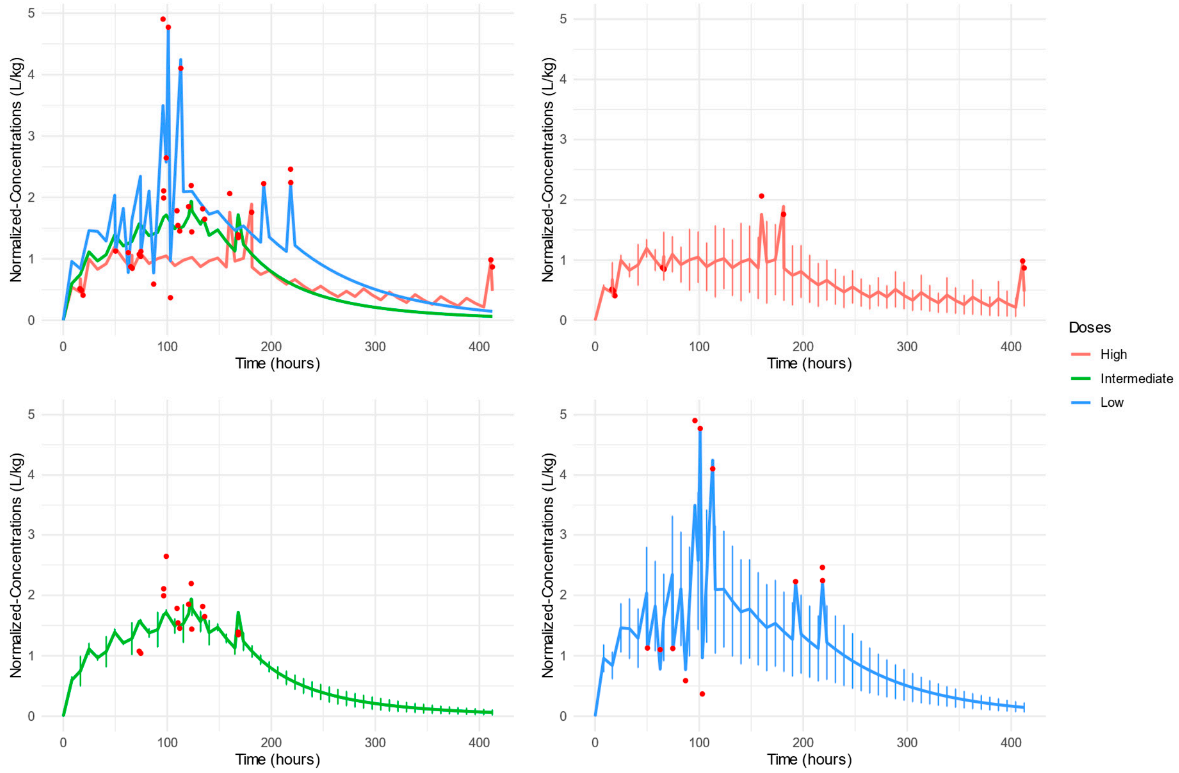

Figure S1. Dose-normalized concentrations for each dose group versus time. Combined dose-normalized concentrations graph (Top left), high doses (9 – 11 mg/kg) graph (Top right), intermediate doses (6 – 8 mg/kg) graph (Bottom left), low doses (3 – 5 mg/kg) graph (Bottom right). Red dots indicate individual dose-normalized concentrations, and whiskers represent standard errors.

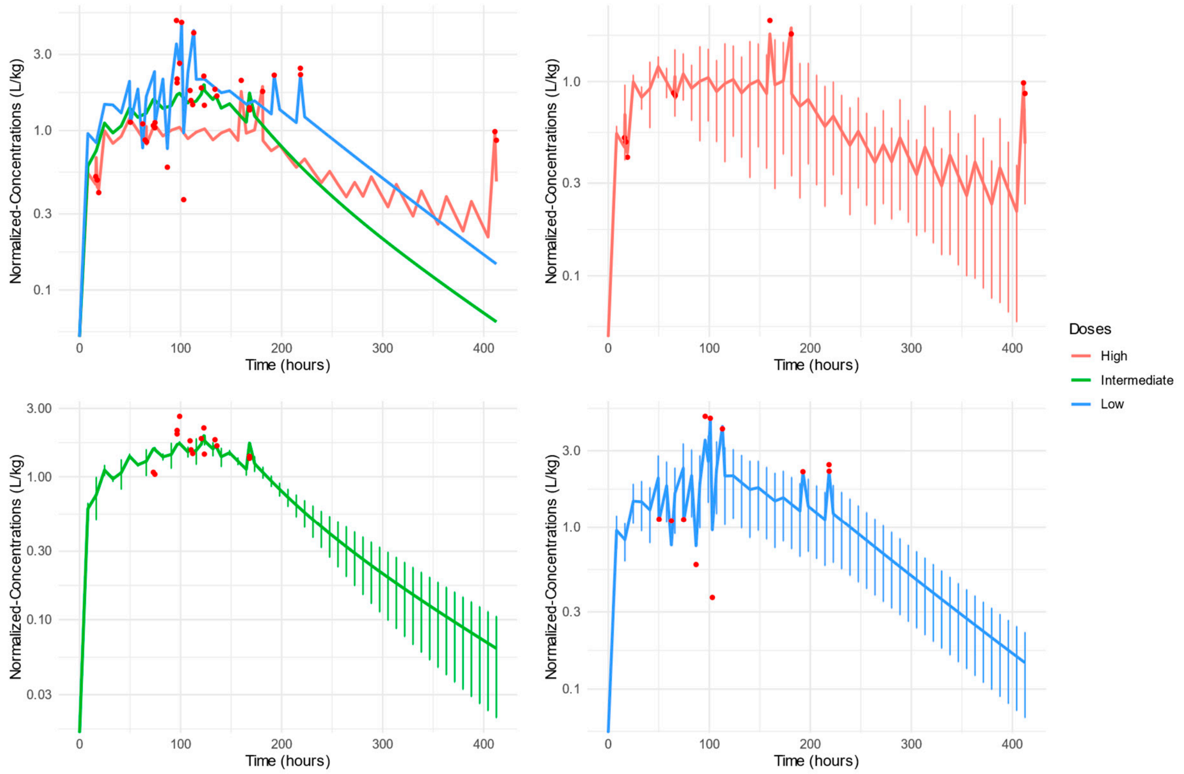

Figure S2. Dose-normalized concentrations (logarithmic scale) for each dose group versus time. Combined dose-normalized concentrations graph (Top left), high doses (9 – 11 mg/kg) graph (Top right), intermediate doses (6 – 8 mg/kg) graph (Bottom left), low doses (3 – 5 mg/kg) graph (Bottom right). Red dots indicate individual dose-normalized concentrations, and whiskers represent standard errors.

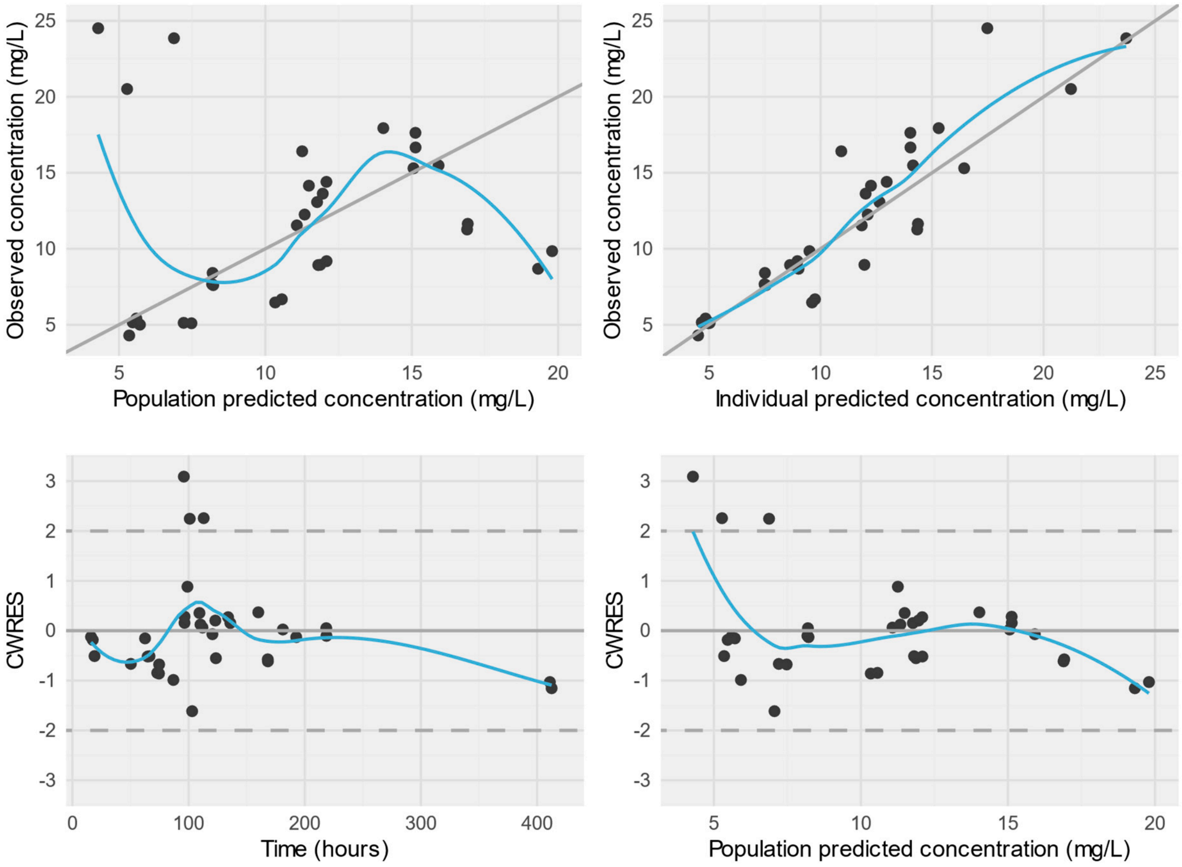

Figure S3. Goodness-of-fit plots of the final fluconazole one-compartment model. Concentration versus population predictions (Top left). Concentration versus individual predictions (Top right). Conditional weighted residuals versus time (Bottom left). Conditional weighted residuals (CWRES) versus population predictions (Bottom right).

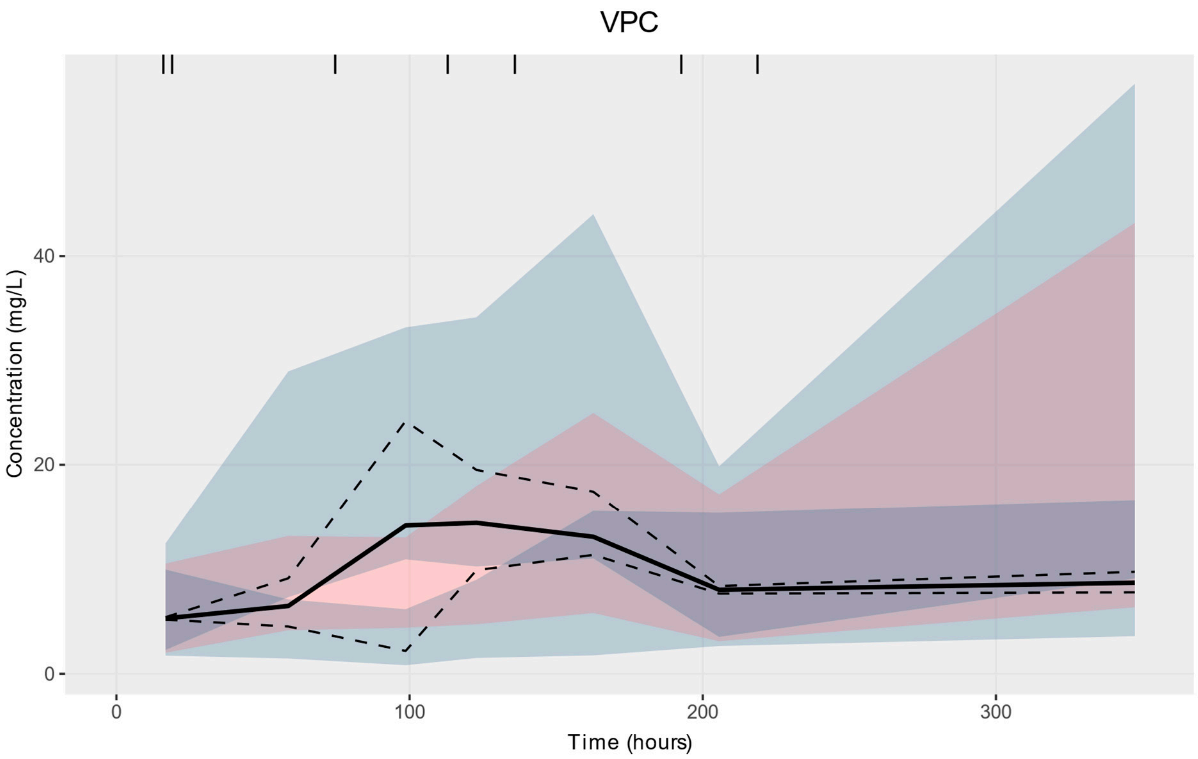

Figure S4. VPC plot of the final fluconazole one-compartment model.

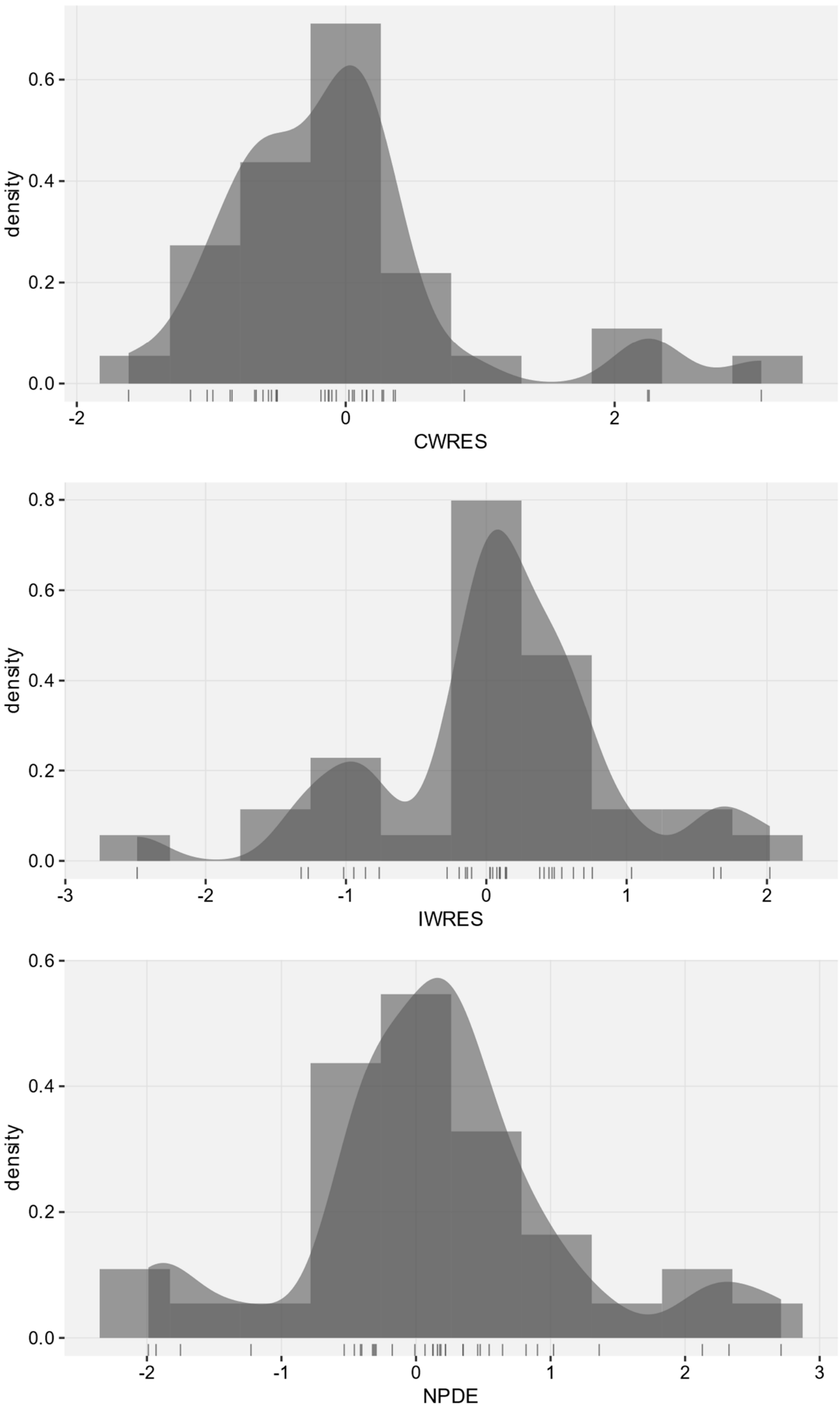

Figure S5. Distribution of residuals.

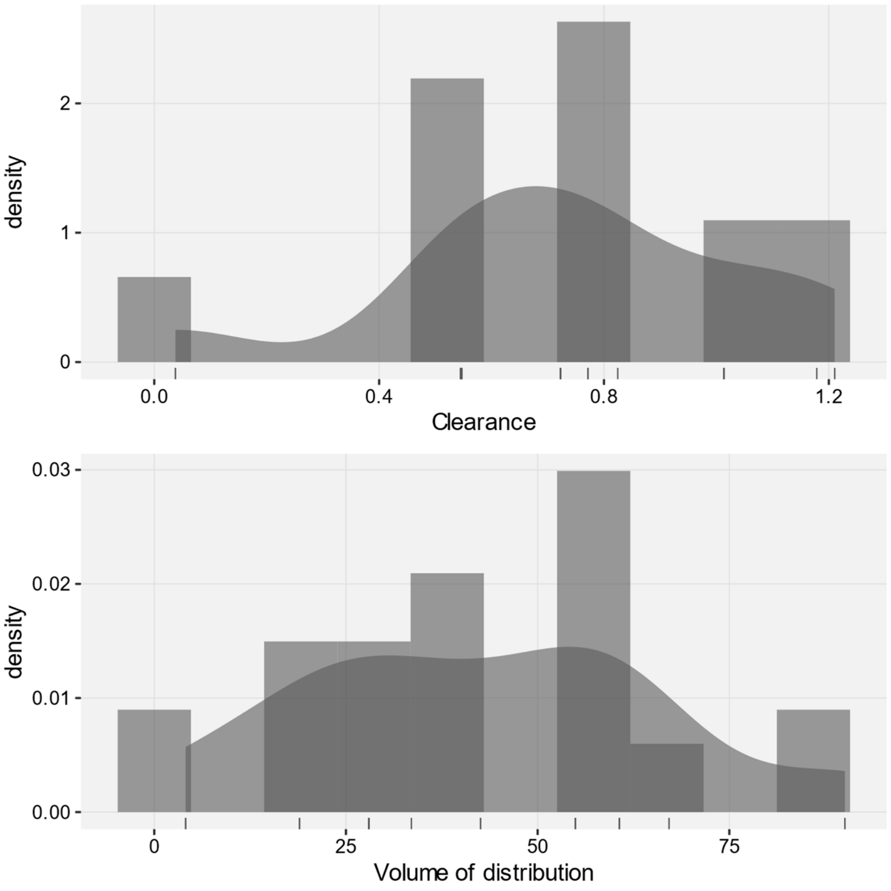

Figure S6. Model parameters distribution.

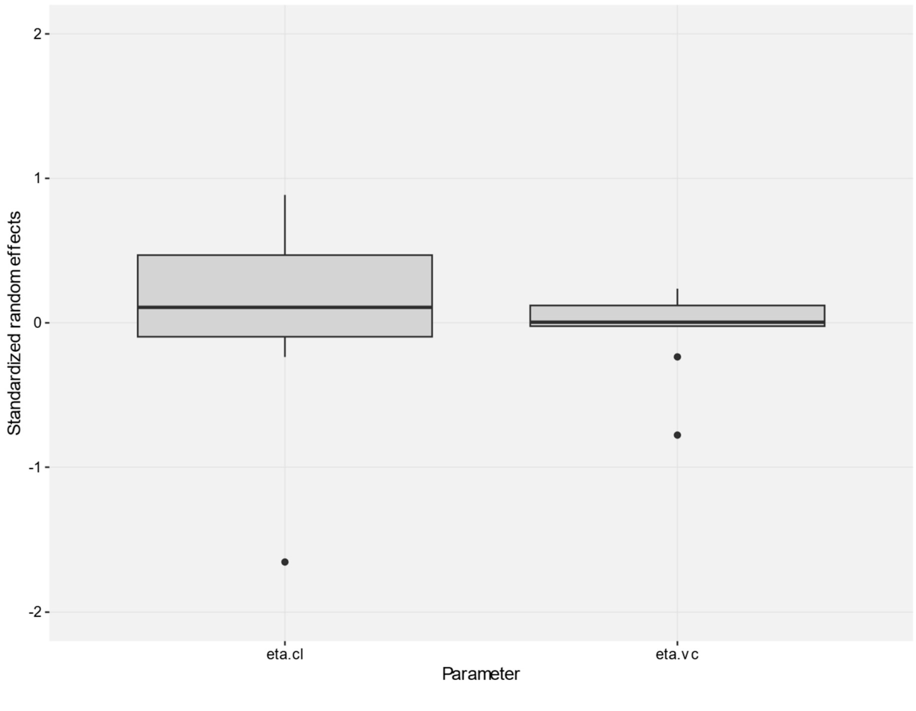

Figure S7. Random effects plots.

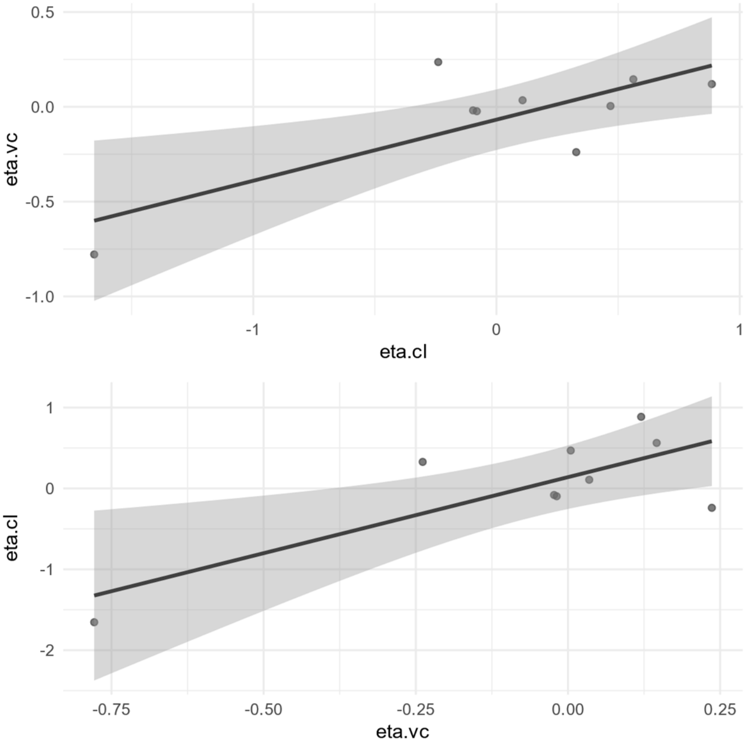

Figure S8. Correlations of random effects.

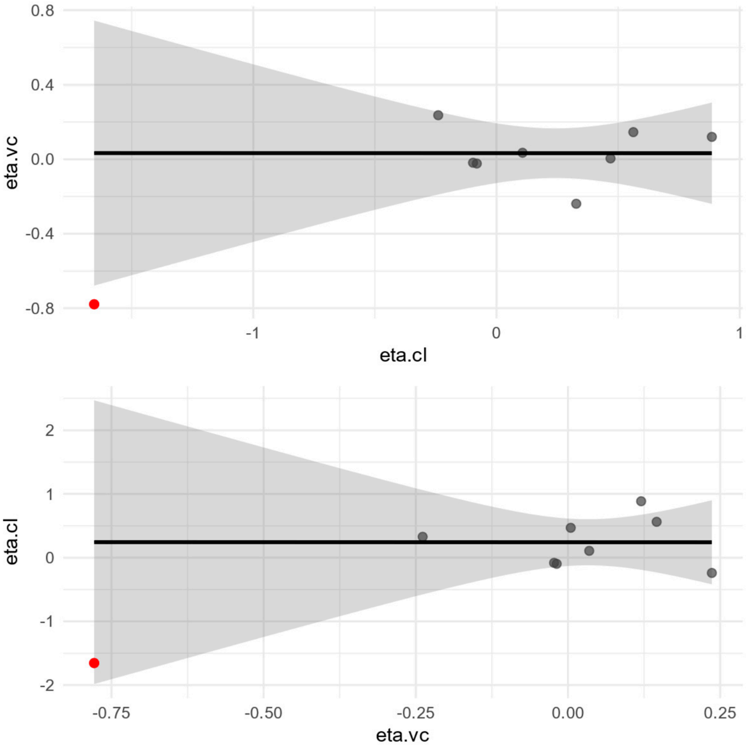

Figure S9. Correlations of random effects excluding outliers (marked in red).

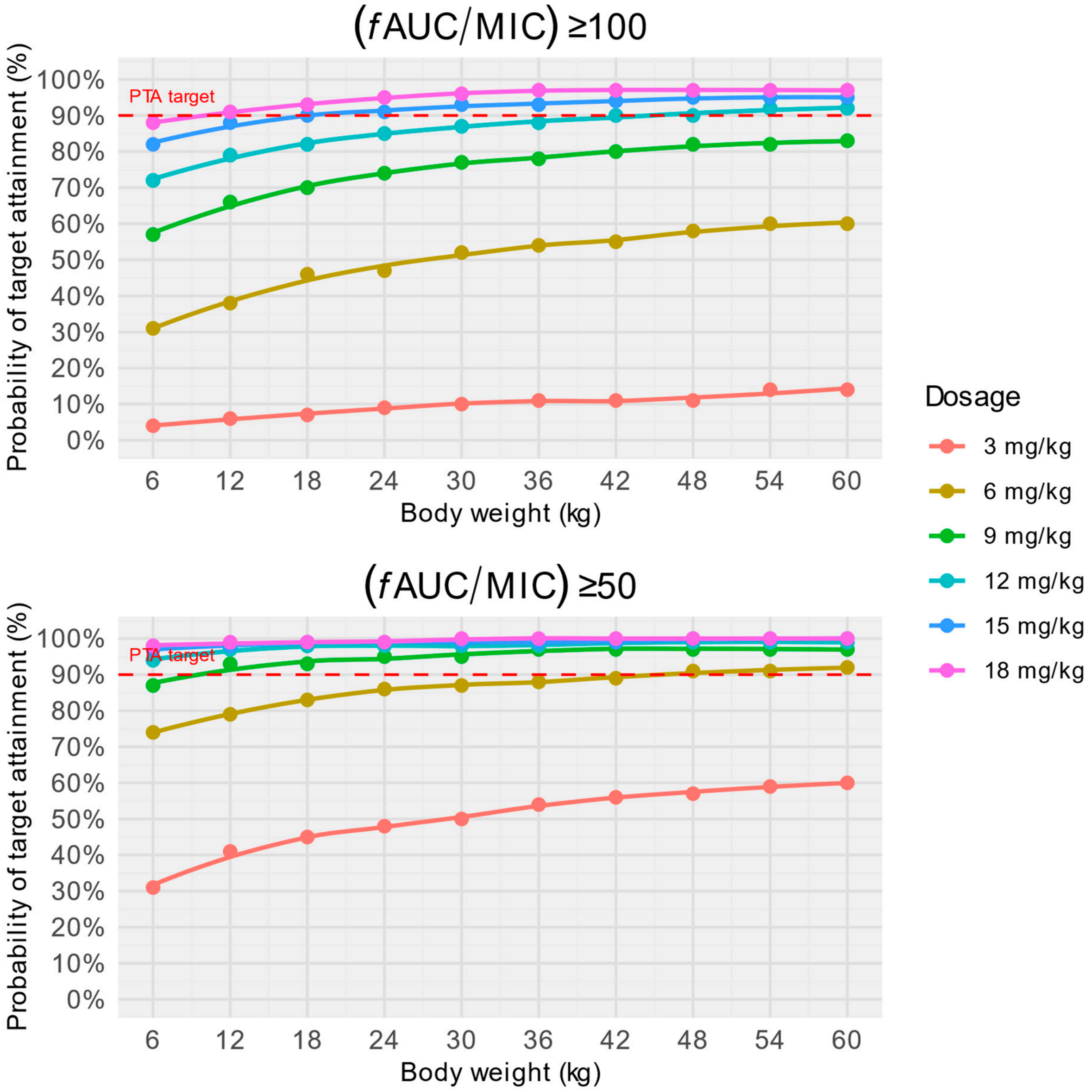

Figure S10. Probability of target attainment charts for fluconazole dosages 3 – 18 mg/kg, body weight 6 – 60 kg, and *f*AUC/MIC ≥ 100 (Top) or *f*AUC/MIC ≥ 50 (Bottom) assuming *Candida* spp MIC = 2 mg/L.

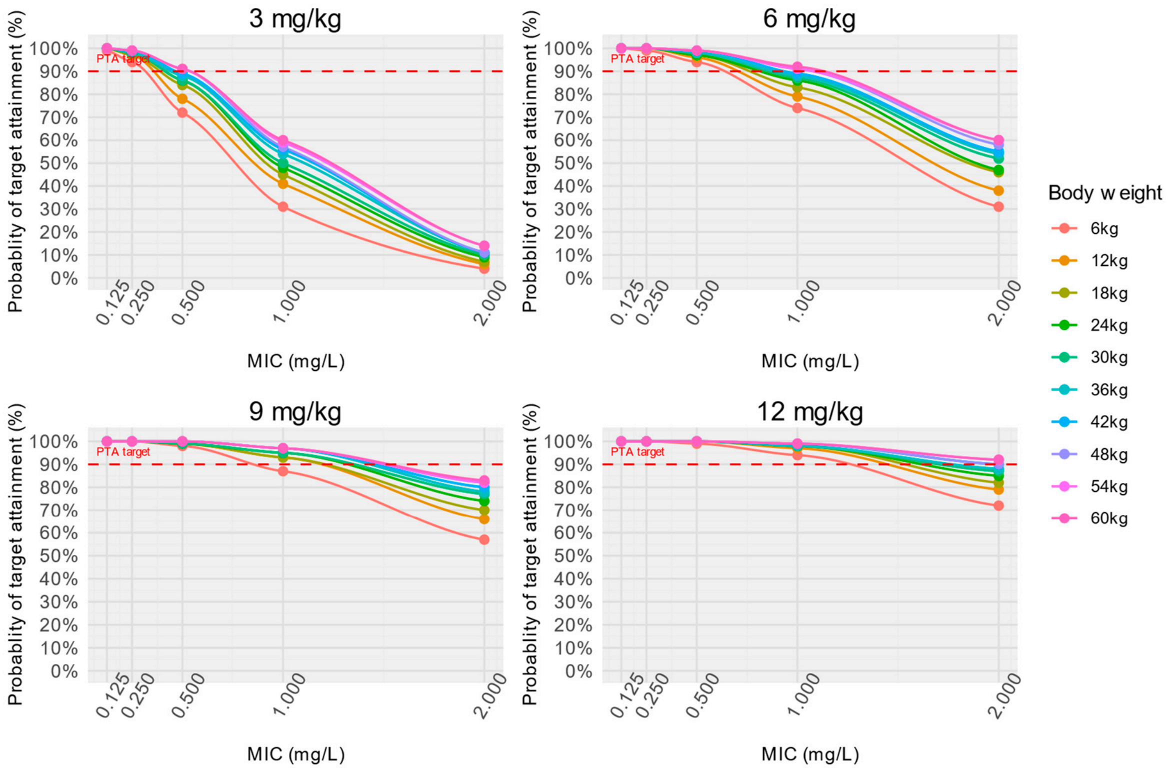

Figure S11. Probability of target attainment charts for fluconazole doses 3 – 12 mg/kg, body weight 6 – 60 kg, and *f*AUC/MIC ≥ 100, assuming different *Candida* spp MICs.

nlmixr2 final model code

````
```{r}

final_model <- function() {
  ini({
    lcl  <- log(1.1)
    lvc   <- log(100)
    prop.err <- 0.25
    eta.cl ~ 0.8
    eta.vc  ~ 0.4
    WT_Cl <- fix(0.75)
    WT_V <- fix(1)
  })
  model({
    cl <- exp(lcl + WT_Cl * log(Weight/70) + eta.cl)
    vc  <- exp(lvc + WT_V * log(Weight/70) + eta.vc)
    d/dt(A_cen) = - cl/vc * A_cen
    cp = A_cen/vc
    cp ~ prop(prop.err)
  })
}

rxClean()
nlmixr2(final_model)

final_run <-
  nlmixr2(
    final_model(),
    data,
    "focei",
    control=list(print=0),
    table=tableControl(cwres = TRUE, npde=TRUE)
  )

final_run
```
````

External hyperlink

The raw dataset and PTA tables with specific % are reachable at the GitHub repository (link below).

https://github.com/arkadiusz-adamiszak/Fluconazole-PopPK.git
